# Sinus-rhythm conduction phenotyping using vector-flow mapping in first-time atrial fibrillation ablation: Associations with structural remodeling and thromboembolic risk

**DOI:** 10.1016/j.hroo.2026.06.012

**Published:** 2026-07-18

**Authors:** Chisato Kitamura, Masatsugu Ohe, Kensuke Hori, Yuichi Hattori, Kosuke Morita, Hideaki Iwahashi, Nagisa Morikawa, Yume Nohara, Jun Kumanomido, Shogo Ito, Yoshihiro Fukumoto

**Affiliations:** Division of Cardiovascular Medicine, Department of Internal Medicine, Kurume University, Fukuoka, Japan

**Keywords:** Atrial fibrillation, Catheter ablation, COHERENT mapping, Conduction phenotypes, Left atrial remodeling, Bachmann’s bundle, Stroke risk, Phenotype burden, Atrial cardiomyopathy

## Abstract

**Background:**

Pulmonary vein isolation achieves success in 60%–80% of first ablation procedures. Sinus-rhythm vector-flow mapping (COHERENT module) may enable phenotype-guided ablation.

**Objective:**

This study aimed to classify sinus-rhythm conduction phenotypes using COHERENT mapping and assess associations with atrial remodeling, recurrence, and thromboembolic risk.

**Methods:**

We prospectively enrolled 80 consecutive patients (mean age 65.1 ± 12.5 years; 32.5% persistent atrial fibrillation [AF]) undergoing initial AF ablation with high-density sinus-rhythm mapping. 5 phenotypes were defined: normal flow, conduction delay, congested flow, disrupted flow, and Bachmann’s bundle split (BBS). Patients were grouped into 8 mutually exclusive, collectively exhaustive phenotype-burden categories (normal, 4 single-phenotype groups, double, triple, quadruple). Outcomes were assessed by unadjusted logistic regression with normal as the reference; all 80 patients completed 24-month follow-up.

**Results:**

Phenotype-burden categories showed gradients in structural and AF-related markers. Each 10 mL/m^2^ left atrial volume index increase was associated with higher odds of double (odds ratio [OR] 2.16), triple (3.02), and quadruple (3.46). 12 of 80 patients (15%) had 24-month recurrence; double showed the only nominally significant association (5 of 15 [33%]; OR 24.62; 95% confidence interval 1.16–521.53). BBS only and quadruple showed borderline odds for previous stroke/transient ischemic attack (each 2 of 7 [28.6%]; OR 21.36).

**Conclusion:**

Sinus-rhythm COHERENT mapping delineated distinct conduction phenotypes associated with progressive atrial structural remodeling. Phenotype burden defined a graded atrial cardiomyopathy continuum, with double showing the highest 24-month recurrence. BBS only and quadruple shared borderline thromboembolic signals via distinct mechanisms. These findings provide mechanistic insight into atrial cardiomyopathy and generate hypotheses for substrate-guided ablation and thromboembolic-risk assessment.

## Graphical abstract

**Figure:**
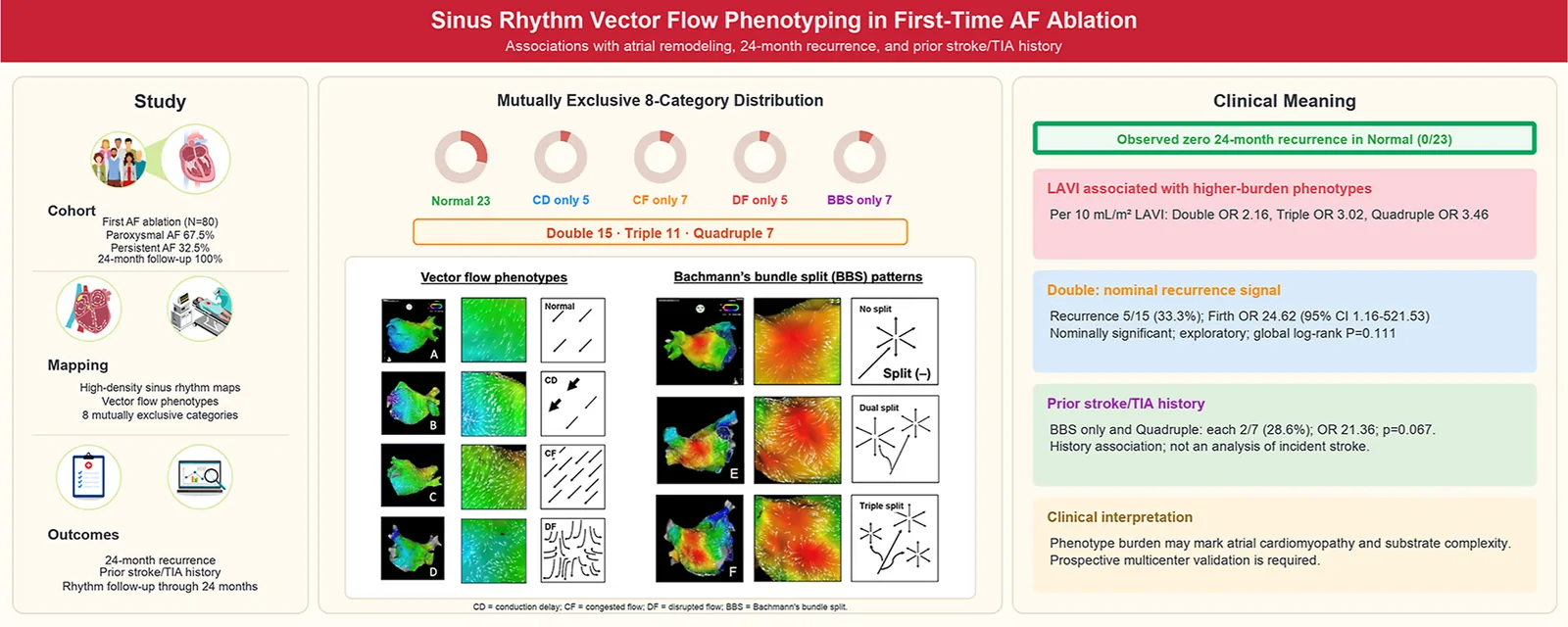

**Key Findings**

▪ In 80 patients undergoing first-time atrial fibrillation ablation, sinus rhythm COHERENT vector flow mapping classified conduction patterns into 8 mutually exclusive phenotype categories defined by phenotype burden—normal, 4 single-phenotype groups (conduction delay only, congested flow only, disrupted flow only, and Bachmann’s bundle split [BBS] only), double, triple, and quadruple—enabling clearer category-stratified comparisons.
▪ Phenotype burden showed graded clinical and remodeling patterns across structural, neurohormonal, and clinical risk markers: left atrial volume index, N-terminal pro-B-type natriuretic peptide, and CHA_2_DS_2_-VASc score all increased progressively from normal to double, triple, and quadruple categories.
▪ During the 24-month follow-up of all 80 patients (100% completeness), although the overall log-rank test was not statistically significant (*P* = .111), the double-phenotype group emerged as the only category showing a nominally significant association with arrhythmia recurrence in the unadjusted Firth model (5 of 15 patients [33.3%]; Firth odds ratio 24.62; 95% confidence interval [CI] 1.16–521.53).
▪ Both BBS-only and quadruple groups showed similarly elevated, borderline odds for previous stroke or transient ischemic attack (each 2 of 7 patients [28.6%]; odds ratio 21.36; 95% CI 0.81–566.30), suggesting that focal Bachmann’s bundle dysfunction and global atrial cardiomyopathy may converge on similar historical thromboembolic association via distinct mechanisms.
▪ The intermediate-substrate double-phenotype group may represent a candidate group for future evaluation of upfront substrate-modifying strategies, whereas BBS-only and quadruple subsets may warrant further study for thromboembolic-risk assessment—findings that require confirmation in larger, multicenter studies.

## Introduction

Pulmonary vein isolation (PVI) remains the cornerstone of catheter ablation for atrial fibrillation (AF), yet the success rate after the first procedure remains 60%–80%, with 20%–40% of patients eventually requiring repeat ablation.^1^, ^2^, ^3^, ^4^ AF progression is linked to electrical and structural atrial remodeling, including interstitial fibrosis and interatrial block, which create complex conduction pathways that persist during sinus rhythm and may contribute to thromboembolic risk.^5^, ^6^, ^7^, ^8^

Conventional bipolar voltage mapping detects scar but provides limited information on wavefront direction or local conduction velocity. Although late gadolinium–enhanced magnetic resonance imaging can quantify atrial fibrosis, a recent randomized trial showed that magnetic resonance imaging–guided fibrosis ablation did not significantly reduce arrhythmia recurrence compared with PVI alone.^9^ These findings highlight the need for alternative substrate characterization approaches.

COHERENT mapping is a vector-flow algorithm that reconstructs continuous vector fields from high-density sinus-rhythm mapping, enabling visualization of conduction delays (CDs), crowding, fragmentation, and split Bachmann’s bundle activation.^10^^,^^11^ Although reports in macroreentrant tachycardia have demonstrated that COHERENT mapping can identify slow conduction isthmuses,^10^ its systematic application in first-time AF ablation remains incompletely examined.^11^

We conducted a prospective cohort study of 80 patients undergoing first-time AF ablation to classify sinus-rhythm conduction into 5 predefined phenotypes—normal flow, CD, congested flow (CF), disrupted flow (DF), and Bachmann’s bundle split (BBS)—and to assess their associations with atrial remodeling, arrhythmia recurrence, and thromboembolic risk.

## Methods

### Study design and oversight

We conducted a prospective, single-center cohort study at Kurume University Hospital. The research reported in this paper adhered to the Declaration of Helsinki as revised in 2013. The protocol was approved by the institutional review board (no. 22280), and all patients gave a written informed consent.

### Patient population

From June 1, 2020, to September 30, 2021, we screened 107 consecutive patients referred for catheter ablation of symptomatic AF. We excluded repeat ablation cases (n = 23) and patients in whom high-density sinus-rhythm mapping was not feasible owing to coronary sinus pacing requirement, which produces a nonphysiological activation pattern bypassing the normal Bachmann’s bundle–mediated interatrial conduction and precludes meaningful interpretation of COHERENT vector-flow patterns (n = 4). The final cohort comprised 80 patients undergoing their first AF ablation (Supplemental Table 1).

Among the 80 patients, 13 (16.3%) had preexisting cardiomyopathy, including tachycardia-induced cardiomyopathy (n = 5), hypertrophic cardiomyopathy (n = 3), dilated cardiomyopathy (n = 2), ischemic cardiomyopathy (n = 1), cardiac amyloidosis (n = 1), and hypertrophic cardiomyopathy with suspected amyloidosis (n = 1). Heart failure was present in 22 patients (27.5%), of whom 10 had underlying cardiomyopathy and 12 had heart failure without cardiomyopathy, predominantly heart failure with preserved ejection fraction.

### Periprocedural management

Oral anticoagulation with vitamin K antagonists was continued, and direct oral anticoagulants were withheld only on the morning of the procedure. Left atrial (LA) thrombus was excluded by transesophageal echocardiography within 24 hours of ablation. Procedures were performed under deep sedation with dexmedetomidine, thiopental, and bolus fentanyl.

### Electroanatomic mapping and phenotype definition

After a single transseptal puncture, a PentaRay catheter (1 mm electrodes; Biosense Webster) was used to acquire high-density sinus-rhythm maps of atria with the CARTO 3 PRIME COHERENT module (software version 7.2). When the intrinsic sinus rate was <50 beats/min, pacing at a cycle length of 600 ms was applied from the high right atrium. LA was mapped until a point confidence score of ≥90% and density of ≥0.5 points/mm^2^ were achieved; datasets failing these criteria were reacquired. Maps contained a median of 1221 points per atrium (interquartile range 933–1527). The COHERENT algorithm reconstructs a continuous vector-flow field in which arrow thickness is inversely related to local conduction velocity.

**Figure 1:**
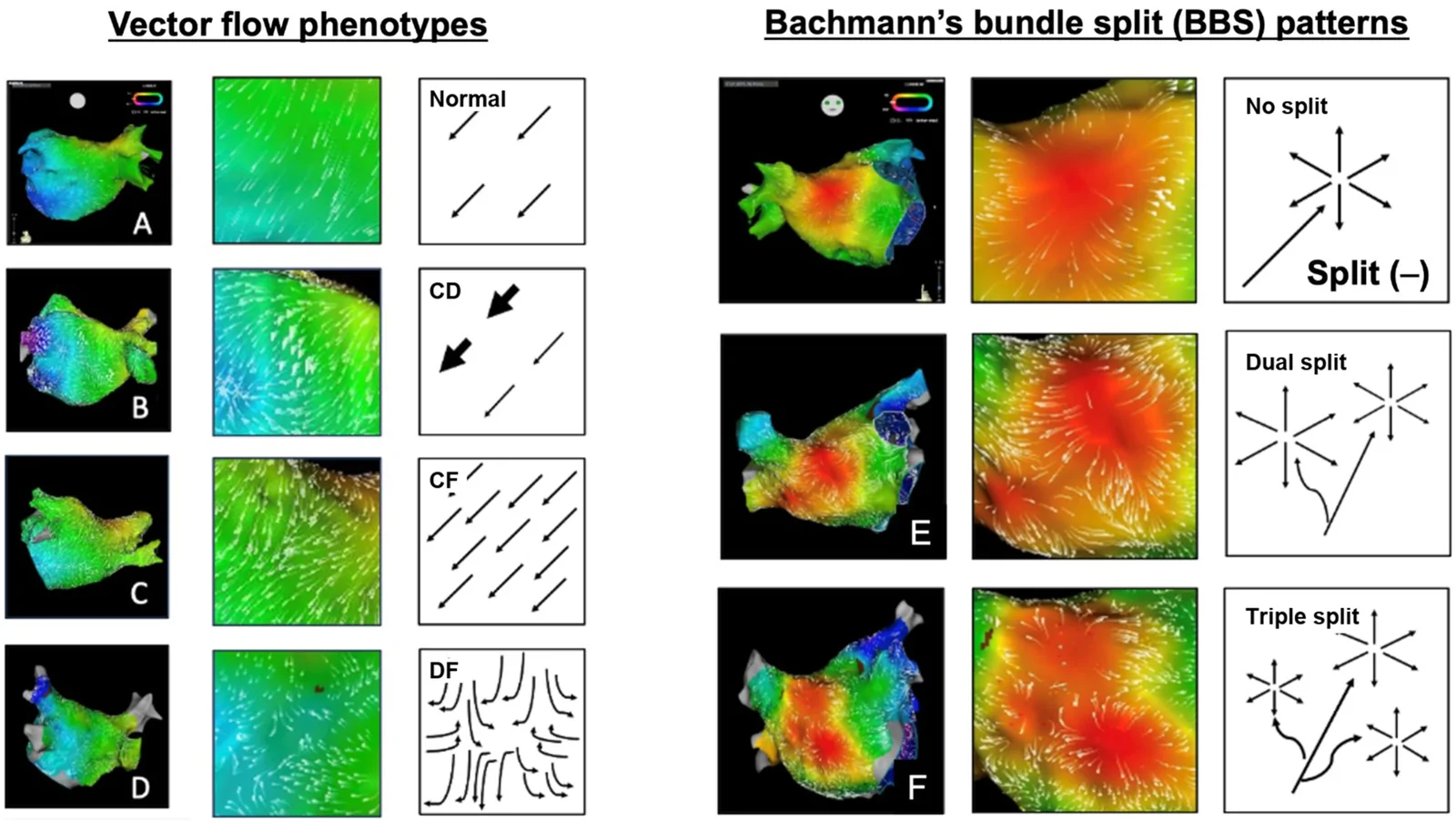
BBS patterns and sinus-rhythm conduction phenotypes illustrated on high-density atrial maps. *Arrows* on each high-density atrial map indicate the instantaneous wavefront direction, with *thicker arrows* signifying slower local conduction (arrow thickness is inversely proportional to conduction velocity). **A–D:** 4 predefined vector-flow conduction phenotypes in sinus rhythm. **A:** Normal flow—uniformly thin, parallel vectors indicating coherent propagation with normal conduction velocity. **B:** CD—globally thickened, uniformly oriented vectors reflecting diffuse conduction slowing. **C:** CF—focal crowding of vectors (a “traffic-jam” pattern) with preserved overall orientation, consistent with a localized conduction bottleneck. **D:** DF—intersecting vectors traveling in 2 or more distinct directions, indicating fragmented wavefront propagation and heterogeneous conduction. *Right:* BBS patterns at the level of Bachmann’s bundle. The top row, labeled “Split (−),” shows single wavefront propagation across Bachmann’s bundle (ie, no BBS), in which a unified wavefront propagates into the left atrium without splitting. BBS is a sinus-rhythm phenotype defined by a single wavefront from the right atrium dividing into multiple divergent wavefronts across Bachmann’s bundle. **E:** Dual split—2 distinct wavefront arms diverging across Bachmann’s bundle. **F:** Triple split—3 separate wavefront arms propagating into the left atrium. BBS = Bachmann’s bundle split; CD = conduction delay; CF = congested flow; DF = disrupted flow.

5 predefined conduction phenotypes were classified based on COHERENT vector-flow patterns (Figure 1): normal flow (uniformly thin, parallel vectors indicating coherent propagation), CD (globally thickened vectors reflecting diffuse slowing), CF (focal crowding of vectors with preserved orientation), DF (intersecting vectors in ≥2 directions indicating fragmented propagation), and BBS (a single right atrial wavefront dividing into multiple divergent wavefronts across Bachmann’s bundle).

2 blinded electrophysiologists independently classified all maps; disagreements (<5%) were resolved by consensus. Each patient could exhibit multiple phenotype components (CD, CF, DF, BBS), which were documented and subsequently combined into the mutually exclusive, collectively exhaustive (MECE) categories described below.

Because phenotype assignments could overlap, patients were grouped into 8 MECE categories defined by the combination of the 4 abnormal phenotypes (CD, CF, DF, BBS): normal (all 4 absent), 4 single-phenotype groups (CD only, CF only, DF only, BBS only), and 3 multiphenotype groups defined by phenotype burden—double (any 2), triple (any 3), and quadruple (all 4). The full 16-category cross-classification of all observed phenotype combinations is presented in Supplemental Table 2.

### Ablation strategy

Wide-area circumferential PVI was performed using a contact-force sensing irrigated catheter (ThermoCool SmartTouch). If CF or DF involved ≥20% of the posterior wall, adjunctive posterior “box” lines were delivered at the operator’s discretion. Acute success required bidirectional block lasting ≥20 minutes.

### Follow-up and clinical endpoints

Antiarrhythmic drugs were discontinued after a 3-month blanking period. Follow-up included 12-lead electrocardiograms (ECGs) at 3, 6, 12, and 24 months at the outpatient clinic and either 24-hour Holter monitoring or ≥7-day patch ECG at 12 months. Additional rhythm monitoring (24-hour Holter, ≥7-day patch ECG, or symptom-driven 12-lead ECG) was performed whenever palpitations or other suggestive symptoms were reported. Implantable loop recorders were not used. All 80 patients completed 24-month follow-up; for 5 patients who could not attend the 24-month clinic visit, recurrence status was ascertained through a structured telephone interview combined with review of any extramural ECG documentation. The primary efficacy endpoint was freedom from any atrial tachyarrhythmia ≥30 seconds beyond the blanking period. Safety endpoints included periprocedural complications, ischemic stroke, and major bleeding.

### Imaging and biomarker assessment

Baseline transthoracic echocardiography measured LA diameter (LAD) (parasternal long-axis view) and indexed LA volume (biplane Simpson’s method). Diastolic function indices (E, A, E/A, and E/e′) were obtained.^12^ Repeat echocardiography at 6 months quantified reverse remodeling as the percentage change in LAD. In the multinomial logistic regression, we prespecified the LA volume index (LAVI) as the primary structural covariate; LAD was used for descriptive trends and for reverse remodeling calculations. Serum N-terminal pro-B-type natriuretic peptide (NT-proBNP) was measured on the morning of the procedure.

### ECG measurements

12-lead ECGs were digitized at 1000 Hz. P-wave duration in lead II was measured from onset to return to the isoelectric line, and the PR interval was measured from P-wave onset to QRS onset. P-wave prolongation was defined as mild (120–139 ms), moderate (140–149 ms), and severe (≥150 ms). Changes at 12 months (ΔP wave and ΔPR) were calculated.

### Statistical analysis

Continuous variables are presented as mean ± standard deviation or median [interquartile range]. Group comparisons used Welch’s *t* test. Categorical variables are summarized as numbers (%), and groups were compared using χ^2^ or Fisher’s exact tests. For phenotype prediction, each candidate predictor was entered individually into a multinomial logistic regression model with the 8 MECE phenotype categories as the outcome and normal as the reference; odds ratios (ORs) with 95% confidence intervals (CIs) are reported. For arrhythmia recurrence and previous stroke/transient ischemic attack (TIA), unadjusted logistic regression with the MECE category as the single predictor and normal as the common reference was applied; because the normal group contained zero events, the Firth penalized likelihood was used to handle zero events in the normal reference group. Arrhythmia-free survival was assessed by Kaplan–Meier analysis with the number at risk displayed beneath each curve. Curves were compared across all 8 categories using the log-rank test; Firth penalized logistic regression used normal as the common reference for category-specific ORs. All analyses were 2 sided, performed using SAS 9.4 (SAS Institute, Cary, NC), and *P* < .05 was considered statistically significant.

To assess the robustness of findings, a sensitivity analysis was performed after excluding 13 patients with preexisting cardiomyopathy, given that atrial cardiomyopathy in these patients may independently affect conduction patterns. Phenotype distribution was also compared between paroxysmal and persistent AF subgroups.

## Results

### Baseline characteristics

The baseline clinical and echocardiographic characteristics of the 80 patients stratified by MECE phenotype category are presented in Table 1. The cohort had a mean age of 65.1 ± 12.5 years with 37 females (46.3%); 54 patients (67.5%) had paroxysmal AF, and 26 (32.5%) persistent AF. Across MECE categories, baseline severity increased with phenotype burden: LAD progressed from 34.5 ± 5.6 mm in normal to 42.1 ± 4.7 mm in quadruple, and CHA_2_DS_2_-VASc score rose from 2.1 ± 1.4 in normal to 3.7 ± 1.5 in quadruple. The prevalence of persistent AF was disproportionately higher in the higher-burden categories (73% in triple, 57% in quadruple) than normal (13%). The overall median preprocedural NT-proBNP concentration was 289 pg/mL [89–661], with marked stratification across categories detailed in Table 2 (2A and 2B).

**Table 1:** Baseline clinical characteristics by MECE phenotype category (N = 80)

Variable	Normal (n = 23)	CD only (n = 5)	CF only (n = 7)	DF only (n = 5)	BBS only (n = 7)	Double (n = 15)	Triple (n = 11)	Quadruple (n = 7)
Age (y)	61.9 ± 15.0	63.2 ± 17.8	70.7 ± 9.0	64.8 ± 4.7	63.4 ± 11.0	67.6 ± 11.8	64.8 ± 10.9	68.1 ± 12.7
Female sex	12 (52.2)	3 (60.0)	4 (57.1)	2 (40.0)	4 (57.1)	5 (33.3)	4 (36.4)	3 (42.9)
Body mass index (kg/m^2^)	23.4 ± 3.3	23.4 ± 3.4	23.3 ± 2.6	26.0 ± 1.2	24.6 ± 3.1	24.2 ± 3.6	25.2 ± 3.9	25.1 ± 7.5
Paroxysmal AF	20 (87.0)	4 (80.0)	5 (71.4)	3 (60.0)	7 (100.0)	9 (60.0)	3 (27.3)	3 (42.9)
Hypertension	10 (43.5)	4 (80.0)	4 (57.1)	2 (40.0)	7 (100.0)	7 (46.7)	8 (72.7)	6 (85.7)
Diabetes mellitus	4 (17.4)	0 (0.0)	0 (0.0)	0 (0.0)	0 (0.0)	1 (6.7)	1 (9.1)	1 (14.3)
Chronic kidney disease	2 (8.7)	1 (20.0)	1 (14.3)	0 (0.0)	0 (0.0)	1 (6.7)	1 (9.1)	1 (14.3)
Heart failure	5 (21.7)	1 (20.0)	2 (28.6)	0 (0.0)	1 (14.3)	6 (40.0)	3 (27.3)	4 (57.1)
Preexisting cardiomyopathy	3 (13.0)	0 (0.0)	1 (14.3)	0 (0.0)	0 (0.0)	3 (20.0)	2 (18.2)	4 (57.1)
Previous stroke/TIA	0 (0.0)	0 (0.0)	0 (0.0)	0 (0.0)	2 (28.6)	0 (0.0)	2 (18.2)	2 (28.6)
CHADS_2_ score	1.0 ± 1.1	1.0 ± 0.7	1.3 ± 1.0	0.4 ± 0.5	2.0 ± 1.2	1.3 ± 1.1	1.6 ± 1.4	2.6 ± 1.0
CHA_2_DS_2_-VASc score	2.1 ± 1.4	2.4 ± 1.7	2.7 ± 1.4	1.0 ± 1.0	2.9 ± 1.1	2.3 ± 1.8	2.6 ± 1.6	3.7 ± 1.5
LVEF (%)	63.6 ± 10.6	68.4 ± 5.7	58.7 ± 18.5	66.0 ± 7.3	69.7 ± 6.7	63.0 ± 9.4	63.5 ± 12.5	62.9 ± 10.3
Left atrial diameter (mm)	34.5 ± 5.6	34.0 ± 6.8	39.7 ± 8.8	40.6 ± 2.2	36.0 ± 4.6	41.2 ± 6.1	42.4 ± 6.9	42.1 ± 4.7
Left atrial volume index (mL/m^2^)∗	28.5 ± 11.4	24.0 ± 5.5	38.3 ± 13.5	37.3 ± 3.3	26.0 ± 6.6	36.5 ± 14.0	40.7 ± 10.9	42.5 ± 7.0
E/A ratio	1.5 ± 1.0	0.9 ± 0.3	1.1 ± 0.1	0.9 ± 0.1	1.0 ± 0.3	1.3 ± 0.6	1.9 ± 1.6	0.9 ± 0.1
E/e′ ratio	8.3 ± 3.6	8.5 ± 1.7	9.0 ± 1.5	7.9 ± 2.1	8.0 ± 1.5	11.9 ± 6.6	10.0 ± 4.0	12.5 ± 4.7
P-wave duration (ms)∗	114 ± 17.7	122 ± 18.9	99 ± 14.0	137 ± 9.2	118 ± 7.2	121 ± 21.4	127 ± 19.5	117 ± 11.7
NT-proBNP (pg/mL)	146 [44–632]	16 [15–125]	269 [167–818]	489 [166–666]	129 [98–355]	288 [123–655]	406 [384–773]	826 [379–2260]

This cohort had a mean age of 65 years and included patients with multiple cardiovascular risk factors (60% hypertension, 27.5% heart failure, median CHA_2_DS_2_-VASc score of 2). Left atrial size was moderately enlarged (mean diameter 38.5 mm). Continuous variables are presented as mean ± standard deviation, categorical variables as n (%), and NT-proBNP as median [interquartile range].

BBS = Bachmann’s bundle split; CD = conduction delay; CF = congested flow; DF = disrupted flow; CHA_2_DS_2_-VASc = congestive heart failure, hypertension, age ≥75 years, diabetes mellitus, stroke/TIA, vascular disease, age 65–74 years, and sex category; LVEF = left ventricular ejection fraction; MECE = mutually exclusive, collectively exhaustive; NT-proBNP = N-terminal pro-B-type natriuretic peptide; TIA = transient ischemic attack.

∗ Missing data: left ventricular ejection fraction in 1 patient (79 evaluable), left atrial diameter in 1 patient (79 evaluable), left atrial volume index in 12 patients (68 evaluable), E/A ratio in 33 patients (47 evaluable), E/e' ratio in 5 patients (75 evaluable), and P-wave duration in 22 patients (58 evaluable).

**Table 2 (A):** Clinical and echocardiographic characteristics by MECE phenotype category (normal and single-phenotype subgroups)

Category	Variable	Normal (n = 23)	CD only (n = 5)	CF only (n = 7)	DF only (n = 5)	BBS only (n = 7)
Demographics	Age, y	61.9 ± 15.0	63.2 ± 17.8	70.7 ± 9.0	64.8 ± 4.7	63.4 ± 11.0
	Female sex	12 (52.2)	3 (60)	4 (57.1)	2 (40)	4 (57.1)
	Body mass index, kg/m^2^	23.4 ± 3.3	23.4 ± 3.4	23.3 ± 2.6	26.0 ± 1.2	24.6 ± 3.1
Atrial fibrillation type	Paroxysmal	20 (87)	4 (80)	5 (71.4)	3 (60)	7 (100)
Comorbidities	Hypertension	10 (43.5)	4 (80)	4 (57.1)	2 (40)	7 (100.0)
	Diabetes mellitus	4 (17.4)	0 (0)	0 (0)	0 (0)	0 (0)
	Chronic kidney disease	2 (8.7)	1 (20)	1 (14.3)	0 (0)	0 (0)
	Sleep apnea	5 (21.7)	1 (20)	0 (0)	2 (40)	0 (0)
	Heart failure	5 (21.7)	1 (20)	2 (28.6)	0 (0)	1 (14.3)
	With cardiomyopathy	3 (13.0)	0 (0)	1 (14.3)	0 (0)	0 (0)
	Previous stroke/TIA	0 (0)	0 (0)	0 (0)	0 (0)	2 (28.6)
Risk scores	CHADS_2_ score	1.0 ± 1.1	1.0 ± 0.7	1.3 ± 1.0	0.4 ± 0.5	2.0 ± 1.2
	CHA_2_DS_2_-VASc score	2.1 ± 1.4	2.4 ± 1.7	2.7 ± 1.4	1.0 ± 1.0	2.9 ± 1.1
Echocardiographic/ECG indices	Left ventricular ejection fraction, %	63.6 ± 10.6	68.4 ± 5.7	58.7 ± 18.5	66.0 ± 7.3	69.7 ± 6.7
	Left atrial diameter, mm	34.5 ± 5.6	34.0 ± 6.8	39.7 ± 8.8	40.6 ± 2.2	36 ± 4.6
	Left atrial volume index, mL/m^2^	28.5 ± 11.4	24.0 ± 5.5	38.3 ± 13.5	37.3 ± 3.3	26.0 ± 6.6
	E/A ratio	1.5 ± 1.0	0.9 ± 0.3	1.1 ± 0.1	0.9 ± 0.1	1.0 ± 0.3
	E/e’ ratio	8.3 ± 3.6	8.5 ± 1.7	9.0 ± 1.5	7.9 ± 2.1	8.0 ± 1.5
	P-wave duration, ms	114 ± 17.7	122 ± 18.9	99 ± 14.0	137 ± 9.2	118 ± 7.2
Biomarker	NT-proBNP, pg/mL	146 [44–632]	16 [15–125]	269 [167–818]	489 [166–666]	129 [98–355]

Values represent number of patients (percentage of subgroup), mean ± standard deviation, or median [interquartile range]. No formal trend test was applied because the 4 single-phenotype subgroups (CD only, CF only, DF only, BBS only) do not have an inherent ordering. Sample sizes are small (n = 5–23), and category comparisons should be interpreted as exploratory.

BBS = Bachmann’s bundle split; CD = conduction delay; CF = congested flow; CHA_2_DS_2_-VASc = congestive heart failure, hypertension, age ≥75 years, diabetes mellitus, stroke/TIA, vascular disease, age 65–74 years, and sex category; DF = disrupted flow; ECG = electrocardiogram; MECE = mutually exclusive, collectively exhaustive; NT-proBNP = N-terminal pro-B-type natriuretic peptide; E/e' = ratio of early diastolic transmitral flow velocity to early diastolic mitral annular velocity; TIA = transient ischemic attack.

**Table 2 (B):** Clinical and echocardiographic characteristics by MECE phenotype category (multiphenotype-burden subgroups)

Category	Variable	Double (n = 15)	Triple (n = 11)	Quadruple (n = 7)	*P* trend
Demographics	Age, yrs	67.6 ± 11.8	64.8 ± 10.9	68.1 ± 12.7	.773
	Female sex	5 (33.3)	4 (36.4)	3 (42.9)	.892
	Body mass index, kg/m^2^	24.2 ± 3.6	25.2 ± 3.9	25.1 ± 7.5	.778
Atrial fibrillation type	Paroxysmal	9 (60)	3 (27.3)	3 (42.9)	.011
Comorbidities	Hypertension	7 (46.7)	8 (72.7)	6 (85.7)	.078
	Diabetes mellitus	1 (6.7)	1 (9.1)	1 (14.3)	.683
	Chronic kidney disease	1 (6.7)	1 (9.1)	1 (14.3)	.922
	Sleep apnea	3 (20)	1 (9.1)	3 (42.9)	.314
	Heart failure	6 (40)	3 (27.3)	4 (57.1)	.398
	With cardiomyopathy	3 (20)	2 (18.2)	4 (57.1)	.091
	Previous stroke/TIA	0 (0)	2 (18.2)	2 (28.6)	.033
Risk scores	CHADS_2_ score	1.3 ± 1.1	1.6 ± 1.4	2.6 ± 1.0	.016
	CHA_2_DS_2_-VASc score	2.3 ± 1.8	2.6 ± 1.6	3.7 ± 1.5	.134
Echocardiographic/ECG indices	Left ventricular ejection fraction, %	63.0 ± 9.4	63.5 ± 12.5	62.9 ± 10.3	.683
	Left atrial diameter, mm	41.2 ± 6.1	42.4 ± 6.9	42.1 ± 4.7	.003
	Left atrial volume index, mL/m^2^	36.5 ± 14.0	40.7 ± 10.9	42.5 ± 7.0	.013
	E/A ratio	1.3 ± 0.6	1.9 ± 1.6	0.9 ± 0.1	.565
	E/e’ ratio	11.9 ± 6.6	10.0 ± 4.0	12.5 ± 4.7	.106
	P-wave duration, ms	121 ± 21.4	127 ± 19.5	117 ± 11.7	.105
Biomarker	NT-proBNP, pg/mL	288 [123–655]	406 [384–773]	826 [379–2260]	.136

Burden-stratified phenotype categories (double, triple, quadruple) demonstrated graded increases in left atrial volume index (LAVI 36.5 → 40.7 → 42.5 mL/m^2^), NT-proBNP (288 → 406 → 826 pg/mL), CHA_2_DS_2_-VASc score (2.3 → 2.6 → 3.7), and previous stroke/TIA prevalence (0% → 18.2% → 28.6%), consistent with a burden-related continuum of atrial cardiomyopathy. *P* values reflect overall group comparison across the 8 MECE categories (analysis of variance for continuous variables; χ^2^ or Fisher’s exact tests for categorical variables). Values are presented as number of patients (percentage of subgroup), mean ± standard deviation, or median [interquartile range].

BBS = Bachmann’s bundle split; CD = conduction delay; CF = congested flow; CHA_2_DS_2_-VASc = congestive heart failure, hypertension, age ≥75 years, diabetes mellitus, stroke/TIA, vascular disease, age 65–74 years, and sex category; DF = disrupted flow; ECG = electrocardiogram; LAVI = left atrial volume index; MECE = mutually exclusive, collectively exhaustive; NT-proBNP = N-terminal pro-B-type natriuretic peptide; E/e' = ratio of early diastolic transmitral flow velocity to early diastolic mitral annular velocity; TIA = transient ischemic attack.

### Distribution of phenotypes

**Figure 2:**
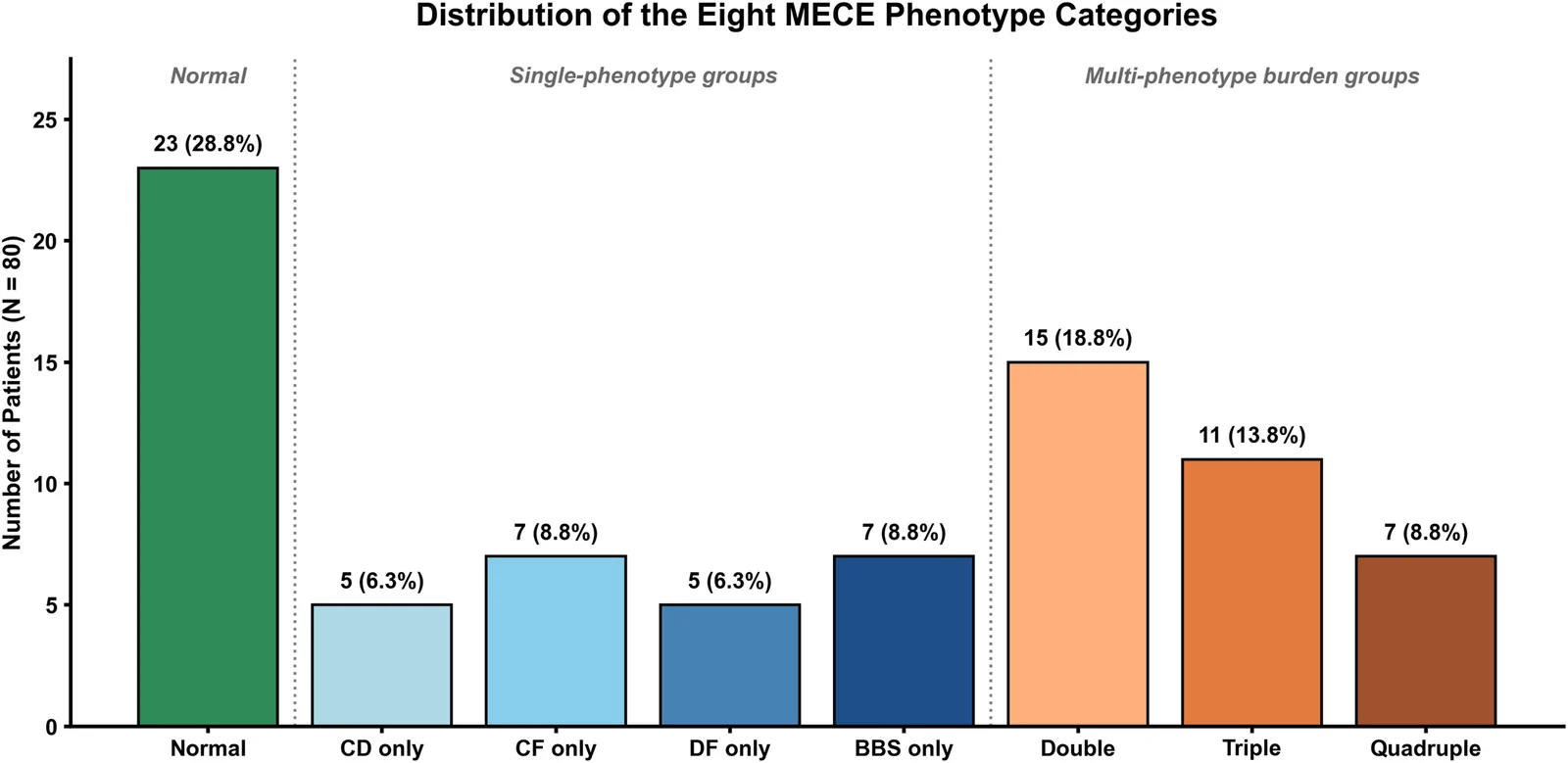
Distribution of the 8 MECE conduction phenotype categories. Patients were classified as normal, single-phenotype categories (CD only, CF only, DF only, BBS only), or multiphenotype-burden categories (double, triple, quadruple) according to the number of abnormal phenotype components present. Colors denote phenotype grouping (normal, *green*; single-phenotype, *blue*; multiphenotype burden, *orange*) and do not represent outcome risk. BBS = Bachmann’s bundle split; CD = conduction delay; CF = congested flow; DF = disrupted flow; MECE = mutually exclusive, collectively exhaustive.

The distribution of conduction phenotypes among the 80 patients in our cohort is presented in Figure 2. Under the MECE classification, the 8 phenotype-burden categories comprised normal in 23 (28.8%), CD only in 5 (6.3%), CF only in 7 (8.8%), DF only in 5 (6.3%), BBS only in 7 (8.8%), double in 15 (18.8%), triple in 11 (13.8%), and quadruple in 7 (8.8%); 33 patients (41.3%) exhibited 2 or more coexisting abnormal phenotypes. Considered individually across isolated and overlapping presentations, CD was observed in 27 patients (33.8%), CF in 36 (45.0%), DF in 27 (33.8%), and BBS in 25 (31.3%). The full 16-category cross-classification is presented in Supplemental Table 2. BBS was absent in all normal-flow patients but coexisted with 1 or more other abnormal phenotypes in 18 of 25 BBS-positive patients (72%).

Abnormal conduction phenotypes were more prevalent in persistent AF than in paroxysmal AF: CF was present in 17 of 26 patients with persistent AF (65.4%) vs 19 of 54 patients with paroxysmal AF (35.2%), DF in 15 (57.7%) vs 12 (22.2%), CD in 15 (57.7%) vs 12 (22.2%), and BBS in 10 (38.5%) vs 15 (27.8%). These findings are consistent with more advanced atrial remodeling in persistent AF (Supplemental Tables 2 and 3).

### Clinical and echocardiographic profiles across conduction phenotypes

Key clinical and echocardiographic variables across the 8 MECE phenotype categories are presented in Table 2 (2A and 2B). LAD and indexed LA volume increased progressively with phenotype burden, from normal (34.5 ± 5.6 mm and 28.5 ± 11.4 mL/m^2^) through single-phenotype groups to double (41.2 ± 6.1 mm; 36.5 ± 14.0 mL/m^2^), triple (42.4 ± 6.9 mm; 40.7 ± 10.9 mL/m^2^), and quadruple (42.1 ± 4.7 mm; 42.5 ± 7.0 mL/m^2^) (*P* for group comparison = .003 and .013, respectively). E/e′ and NT-proBNP showed numerically similar gradients (normal E/e′ 8.3 vs quadruple 12.5; normal NT-proBNP median 146 pg/mL [44–632] vs quadruple 826 pg/mL [379–2260]), although these trends did not reach statistical significance (*P* = .106 and *P* = .136). Surface P-wave duration also did not differ significantly across categories (*P* = .105). The prevalence of preexisting cardiomyopathy rose from 13% in normal to 57% in quadruple (*P* for group comparison = .091).

### Phenotype-specific clinical correlates

In multinomial logistic regression with normal as the reference (Table 3), each candidate predictor was assessed individually. The LAVI showed a graded dose-response across phenotype burden: each 10 mL/m^2^ increase was associated with significantly higher odds of CF only (OR 2.51 [95% CI 1.02–6.14]), double (OR 2.16 [1.03–4.53]), triple (OR 3.02 [1.29–7.09]), and quadruple (OR 3.46 [1.34–8.97]), with nonsignificant point estimates for CD only, DF only, and BBS only. Each doubling in NT-proBNP was significantly associated with triple (OR 1.66 [1.07–2.59]) and quadruple (OR 2.02 [1.16–3.51]) and inversely with CD only (OR 0.51 [0.27–0.99]). Persistent (vs paroxysmal) AF showed the strongest associations with the highest-burden categories, with markedly elevated odds for triple (OR 17.78 [2.94–107.35]) and quadruple (OR 8.89 [1.29–61.06]). Additional predictors (P-wave duration, CHA_2_DS_2_-VASc score, age, sex, and body mass index) are presented in Supplemental Table 4.

**Table 3:** Single-predictor multinomial logistic regression: clinical predictors of MECE conduction phenotype categories (vs normal)

Phenotype (vs normal)	LAVI (per 10 mL/m^2^)	NT-proBNP (per doubling)	Persistent AF
CD only	0.57 (0.15–2.12)	0.51 (0.27–0.99)	1.67 (0.14–20.40)
CF only	2.51 (1.02–6.14)	1.50 (0.91–2.47)	2.67 (0.35–20.51)
DF only	2.30 (0.79–6.70)	1.34 (0.78–2.33)	4.44 (0.51–38.61)
BBS only	0.75 (0.28–2.00)	1.01 (0.64–1.60)	NE
Double	2.16 (1.03–4.53)	1.27 (0.88–1.83)	4.44 (0.90–21.87)
Triple	3.02 (1.29–7.09)	1.66 (1.07–2.59)	17.78 (2.94–107.35)
Quadruple	3.46 (1.34–8.97)	2.02 (1.16–3.51)	8.89 (1.29–61.06)

Estimates are unadjusted ORs with 95% CIs from multinomial logistic regression with the 8 MECE phenotype categories as the outcome (normal as the reference) and each predictor entered 1 at a time. Statistically significant associations (*P* < .05) include LAVI (per 10 mL/m^2^) with CF only (OR 2.51), double (OR 2.16), triple (OR 3.02), and quadruple (OR 3.46); NT-proBNP (per doubling) with triple (OR 1.66) and quadruple (OR 2.02); and persistent AF with triple (OR 17.78) and quadruple (OR 8.89). LAVI was missing in 12 of the 80 patients (68 evaluable). Additional predictors (P-wave duration, CHA_2_DS_2_-VASc score, age, sex, body mass index) are presented in Supplemental Table 4.

AF = atrial fibrillation; BBS = Bachmann’s bundle split; CD = conduction delay; CF = congested flow; CHA_2_DS_2_-VASc = congestive heart failure, hypertension, age ≥75 years, diabetes mellitus, stroke/TIA, vascular disease, age 65–74 years, and sex category; CI = confidence interval; DF = disrupted flow; LAVI = left atrial volume index; MECE = mutually exclusive, collectively exhaustive; NE = not estimable (zero events in that cell); NT-proBNP = N-terminal pro-B-type natriuretic peptide; OR = odds ratio.

### Arrhythmia recurrence and reverse remodeling

**Figure 3:**
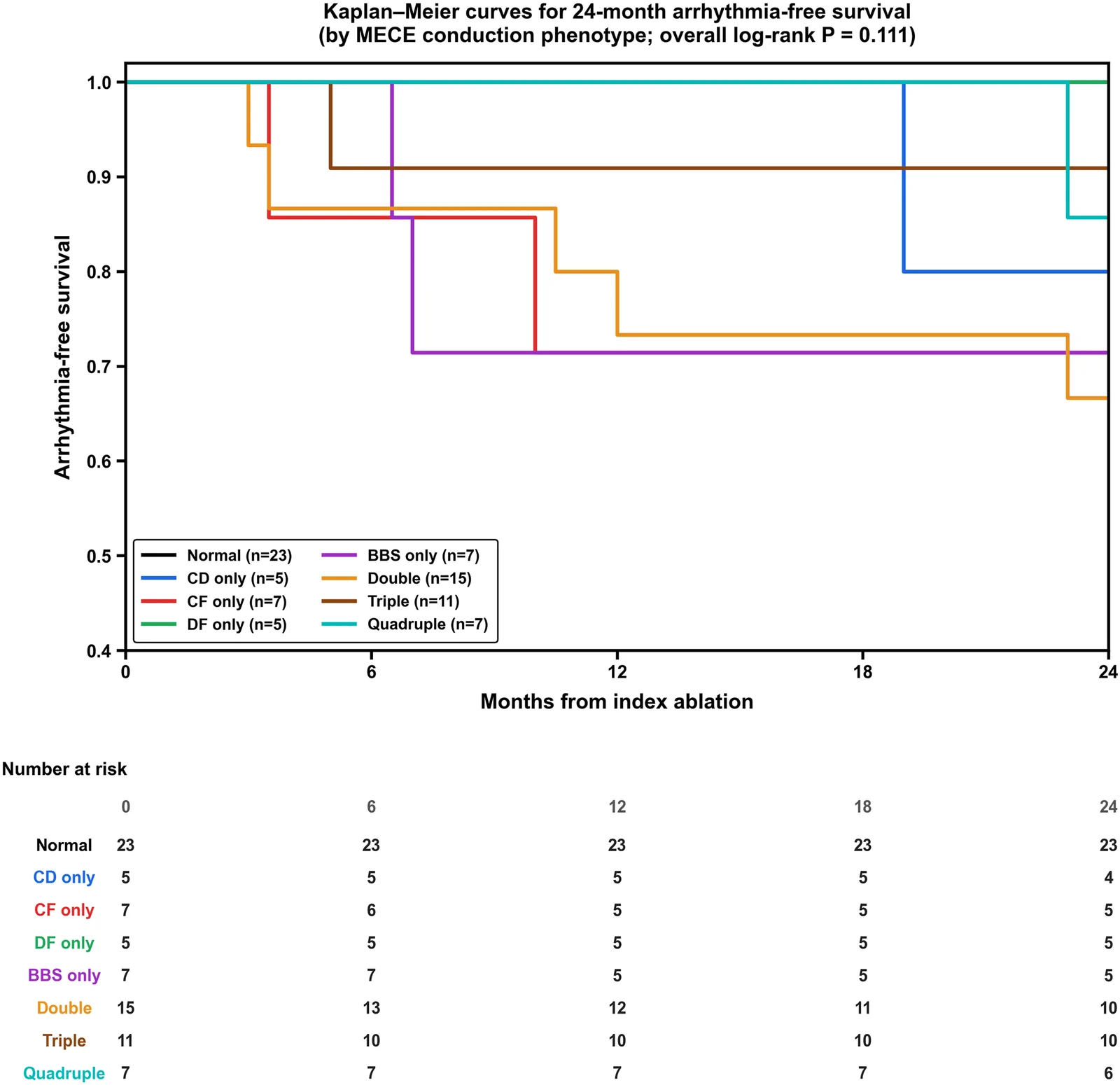
24-month arrhythmia-free survival by MECE conduction phenotype categories (exploratory). Kaplan–Meier curves showing arrhythmia-free survival over 24 months stratified by the 8 MECE conduction phenotype categories; the corresponding Firth analyses (Table 4) use normal as the common reference. The number at risk at each time point is shown below the time axis. The overall log-rank test was not statistically significant (*P* = .111). Detailed Firth penalized odds ratios for each MECE category vs normal are presented in Table 4. Category-specific estimates are exploratory and were not corrected for multiple comparisons. BBS = Bachmann’s bundle split; CD = conduction delay; CF = congested flow; CI = confidence interval; DF = disrupted flow; MECE = mutually exclusive, collectively exhaustive; OR = odds ratio.

At 24 months, 12 of 80 patients (15.0%) experienced arrhythmia recurrence, with no recurrence within the 3-month blanking period. Kaplan–Meier curves of arrhythmia-free survival stratified by the 8 MECE phenotype categories are presented in Figure 3; the corresponding Firth analyses (Table 4) used normal as the common reference. The overall log-rank test did not reach statistical significance (*P* = .111). Recurrence rates by category were normal 0 of 23 (0.0%), CD only 1 of 5 (20.0%), CF only 2 of 7 (28.6%), DF only 0 of 5 (0.0%), BBS only 2 of 7 (28.6%), double 5 of 15 (33.3%), triple 1 of 11 (9.1%), and quadruple 1 of 7 (14.3%). In unadjusted Firth penalized logistic regression with normal as the reference (Table 4), the double category was the only category showing a nominally significant association with 24-month recurrence (OR 24.62 [95% CI 1.16–521.53]; *P* = .040). CF only and BBS only showed borderline elevations (each OR 21.37 [0.81–566.33]; *P* = .067), and the remaining categories (CD only 15.67; DF only 4.27; triple 6.71; quadruple 10.85) were not significant. LA reverse remodeling at 6 months remained modest across categories and was driven primarily by baseline LAD. The 12-month secondary analysis is presented in Supplemental Table 5.

**Table 4:** 24-month arrhythmia recurrence by MECE conduction phenotype category

Phenotype	n	Events (%)	Firth OR (95% CI)	*P* value
Normal (reference)	23	0 (0.0)	Reference	—
CD only	5	1 (20.0)	15.67 (0.47–521.45)	.124
CF only	7	2 (28.6)	21.37 (0.81–566.33)	.067
DF only	5	0 (0.0)	4.27 (0.06–306.01)	.505
BBS only	7	2 (28.6)	21.37 (0.81–566.33)	.067
Double	15	5 (33.3)	24.62 (1.16–521.53)	.040∗
Triple	11	1 (9.1)	6.71 (0.23–195.91)	.269
Quadruple	7	1 (14.3)	10.85 (0.35–336.09)	.174

Data are unadjusted ORs with 95% CIs from Firth penalized logistic regression with the 8 MECE phenotype categories as the predictor and 24-month arrhythmia recurrence as the outcome (normal as the reference). 12 patients (15.0%) experienced arrhythmia recurrence over the 24-month follow-up period; all 80 patients completed follow-up (zero lost to follow-up). No recurrences occurred during the 3-month blanking period. The double-phenotype category was the only group nominally associated with recurrence in the unadjusted Firth model (Firth OR 24.62; 95% CI 1.16–521.53; *P* = .040), although the global log-rank test across the 8 categories did not reach statistical significance (*P* = .111). The Firth penalized likelihood was used because the normal reference group had zero events. The 12-month version of this analysis is presented in Supplemental Table 5 for comparison. Category-specific estimates are exploratory and were not corrected for multiple comparisons.

BBS = Bachmann’s bundle split; CD = conduction delay; CF = congested flow; CI = confidence interval; DF = disrupted flow; MECE = mutually exclusive, collectively exhaustive; OR = odds ratio.

∗ Statistically significant.

### History of stroke/TIA and phenotype associations

Six patients (7.5%) had a history of stroke or TIA, distributed across 3 of the 8 MECE categories: BBS only (2 of 7 [28.6%]), triple (2 of 11 [18.2%]), and quadruple (2 of 7 [28.6%]). The remaining 5 categories contained no stroke/TIA events. In unadjusted Firth penalized logistic regression with normal as the reference (Table 5), none of the categories reached statistical significance, although BBS only and quadruple showed borderline elevations of identical magnitude (each OR 21.36 [95% CI 0.81–566.30]; *P* = .067), and triple showed a nonsignificant elevation (OR 12.37 [0.50–306.52]; *P* = .125). These associations are exploratory given the small number of events (n = 6) and very wide CIs.

**Table 5:** Association between MECE conduction phenotype categories and history of stroke/TIA

Phenotype	n	Events (%)	Firth OR (95% CI)	*P* value
Normal (reference)	23	0 (0.0)	Reference	—
CD only	5	0 (0.0)	4.27 (0.06–306.03)	.505
CF only	7	0 (0.0)	3.13 (0.05–206.80)	.593
DF only	5	0 (0.0)	4.27 (0.06–306.03)	.505
BBS only	7	2 (28.6)	21.36 (0.81–566.30)	.067
Double	15	0 (0.0)	1.52 (0.03–89.68)	.842
Triple	11	2 (18.2)	12.37 (0.50–306.52)	.125
Quadruple	7	2 (28.6)	21.36 (0.81–566.30)	.067

Data are unadjusted ORs with 95% CIs from Firth penalized logistic regression with the 8 MECE phenotype categories as the predictor and history of stroke or TIA as the outcome (normal as the reference). 6 patients (7.5%) had a history of stroke or TIA. 2 categories—BBS only and quadruple—showed identical borderline elevations (each 2 of 7 patients; OR 21.36; 95% CI 0.81–566.30; *P* = .067), consistent with 2 distinct mechanisms (focal interatrial block vs advanced atrial cardiomyopathy) converging on similar thromboembolic risk. The Firth penalized likelihood was used because the normal reference group had zero events.

BBS = Bachmann’s bundle split; CD = conduction delay; CF = congested flow; CI = confidence interval; DF = disrupted flow; MECE = mutually exclusive, collectively exhaustive; OR = odds ratio; TIA = transient ischemic attack.

### Procedural characteristics and adjunctive lesions

Wide-area circumferential PVI was successfully achieved in all patients. No procedure-related complications or thromboembolic/bleeding events occurred. A posterior wall “box” isolation was performed in 8 of 80 procedures (10%) without significant differences among phenotypes. Additional lesion sets such as complex fractionated atrial electrogram ablation, cavotricuspid isthmus ablation, or superior vena cava isolation were applied at the operator’s discretion based on substrate complexity. The proportion of patients receiving any adjunctive substrate-modifying lesion beyond PVI increased with phenotype burden: 20% in double, 36% in triple, and 57% in quadruple.

In a sensitivity analysis excluding 13 patients with preexisting cardiomyopathy (n = 67), patients with cardiomyopathy were disproportionately represented among abnormal phenotypes (CF 76.9%; DF 61.5%). After their exclusion, the associations between DF and structural remodeling markers remained consistent: LAVI was 40.1 ± 7.3 mL/m^2^ in DF vs 29.4 ± 11.1 mL/m^2^ in non-DF patients, and NT-proBNP was 681 ± 692 pg/mL vs 301 ± 359 pg/mL. The association between BBS and previous stroke/TIA also persisted, with 4 of 20 BBS patients (20.0%) having a history of stroke/TIA compared with 0 of 47 non-BBS patients (0%). Recurrence patterns were similar to those observed in the full cohort (Supplemental Table 6).

## Discussion

This study applied COHERENT vector-flow mapping to characterize sinus-rhythm conduction patterns in patients undergoing first-time AF ablation, and examined their associations with atrial remodeling, recurrence, and thromboembolic risk. Several key findings warrant discussion.

First, we observed a graded biological gradient across the 8 MECE phenotype categories, consistent with phenotype burden representing a continuum of progressive atrial cardiomyopathy. LAVI, NT-proBNP, and CHA_2_DS_2_-VASc score all increased progressively with phenotype burden, culminating in the quadruple group (LAVI 42.5 mL/m^2^, NT-proBNP 826 pg/mL, preexisting cardiomyopathy in 57%). In multinomial logistic regression (Table 3), each 10 mL/m^2^ increase in LAVI was significantly associated with higher odds of double (OR 2.16), triple (OR 3.02), and quadruple (OR 3.46); each doubling in NT-proBNP was significantly associated with triple (OR 1.66) and quadruple (OR 2.02); and persistent AF was strongly linked to the highest-burden categories (triple OR 17.78; quadruple OR 8.89). In addition, quadruple-phenotype membership was associated with an elevated CHA_2_DS_2_-VASc score (OR 2.06; 95% CI 1.11–3.84, per 1 point increase), reflecting accumulated systemic thromboembolic risk. These findings are consistent with previous studies showing that atrial enlargement and elevated natriuretic peptide levels are markers of atrial cardiomyopathy and predictors of AF recurrence after ablation; meta-analyses have demonstrated that each 1 mm increase in LAD and 1 mL/m^2^ increase in LAVI raise recurrence risk by 12% and 2%, respectively.^8^^,^^13^, ^14^, ^15^ Our work extends this concept by linking structural enlargement and neurohormonal activation to the burden of abnormal sinus-rhythm conduction phenotypes within a unifying atrial cardiomyopathy framework.

Adjunctive ablation rates increased with phenotype burden (20% in double, 36% in triple, 57% in quadruple), suggesting that operator decision making routinely escalates ablation strategy with increasing substrate complexity. In our Japanese clinical practice, additional substrate-modifying ablation—including posterior wall “box” isolation, complex fractionated atrial electrogram defragmentation, cavotricuspid isthmus ablation, and superior vena cava isolation—is performed at the first ablation procedure when substrate complexity warrants it, in contrast to the predominantly pulmonary-vein-isolation-only strategy adopted in many trials conducted outside Japan. The relatively conservative adjunctive ablation in the double group, combined with its highest recurrence rate, supports the hypothesis that this intermediate substrate population may particularly benefit from more aggressive upfront substrate-modifying strategies. CF only and BBS only also showed borderline elevated recurrence (each 2 of 7 [28.6%]; OR 21.37 [0.81–566.33]; *P* = .067); CF was characterized by preserved conduction velocity with focal vector crowding and elevated diastolic filling pressures, features aligning with diastolic dysfunction.^12^^,^^16^ Therefore, in our cohort, CF only may represent a pressure-overload atrial myopathy rather than diffuse fibrosis. Notably, the double category includes patients with CF as 1 of 2 coexisting features, suggesting that similar pressure-overload mechanisms may contribute to the recurrence signal observed in both CF-only and double phenotypes.

Whether ablation beyond PVI improves outcomes in patients with the double or CF only phenotypes remains to be determined in adequately powered studies. Management of diastolic dysfunction and hypertension management may also warrant investigation as adjunctive strategies.^17^

Patients with impaired diastolic function, reflected by a high E/e′ ratio, have a markedly higher risk of AF recurrence; an E/e′ of ≥13.45 has been shown to triple the hazard of late recurrence. In addition, slow LA conduction velocity (<0.80 m/s) has been shown to be a strong predictor of post-ablation recurrence.^18^ A prospective study using COHERENT mapping similarly identified an anterior LA conduction velocity of <0.83 m/s as a strong independent predictor of AF recurrence after PVI.^19^ Similarly, LA strain—an echocardiographic measure of LA function—has been reported as a predictor of paroxysmal AF recurrence.^20^ Elevated NT-proBNP, a marker of atrial myocyte stress, also predicts recurrence: preablation NT-proBNP levels of ≥168 pg/mL nearly triple the risk.^21^ Furthermore, in patients with persistent AF, a greater 1-month reduction in NT-proBNP after ablation has been associated with a lower AF recurrence rate.^22^

Third, 2 distinct phenotype categories—BBS only and quadruple—showed identical borderline elevations in stroke/TIA history (each 2 of 7 patients [28.6%]; OR 21.36 [95% CI 0.81–566.30]; *P* = .067), with triple also showing a nonsignificant signal (2 of 11 [18.2%]; OR 12.37 [0.50–306.52]; *P* = .125). We interpret this pattern as a dual-mechanism convergence on thromboembolic vulnerability: BBS only represents a focal interatrial conduction disorder anchored at Bachmann’s bundle, whereas quadruple represents a global atrial cardiomyopathy with the largest LA dimensions and highest natriuretic peptide levels. That 2 distinct substrate phenotypes—1 focal and 1 global—reach the same magnitude of stroke/TIA association suggests that thromboembolic vulnerability in this cohort may emerge through more than 1 pathway. The BBS-only observation is conceptually consistent with Bayés syndrome, in which interatrial block manifests as a prolonged P wave and is associated with stroke and AF.^23^, ^24^, ^25^ However, advanced interatrial block was not formally defined by ECG criteria in this study, and the wide CIs reflect the small number of events (n = 6); these associations should therefore be regarded as hypothesis-generating. Epicardial mapping studies have shown that conduction disorders across Bachmann’s bundle are more common in patients with AF and structural heart disease.^26^^,^^27^ Such conduction abnormalities have been consistently shown to increase vulnerability to AF.^28^ Whether COHERENT mapping identifies patients who might benefit from closer thromboembolic-risk assessment warrants investigation in larger cohorts.

Individual phenotype effects may differentiate more clearly when these phenotypes coexist (as in double, triple, or quadruple) than when they occur in isolation, suggesting that phenotype combinations rather than single patterns may better characterize the recurrence-relevant substrate. Larger, multicenter cohorts will be required to permit robust phenotype-specific inferences, and detailed phenotype-level analyses will require larger sample sizes. The consistently elevated estimates for the double-phenotype group across multiple analyses nonetheless support a real association rather than a random finding.

Importantly, a sensitivity analysis excluding patients with preexisting cardiomyopathy supported the consistency of the key findings. The progressive structural gradient across phenotypes and the association between BBS and previous stroke/TIA were preserved after removing patients in whom atrial conduction abnormalities might be attributable to the underlying cardiomyopathy rather than to AF-related remodeling. The higher prevalence of abnormal phenotypes in persistent AF than paroxysmal AF further supports the biological plausibility of the phenotypic classification, given that persistent AF is associated with more advanced atrial substrate.

An exploratory subgroup analysis (Supplemental Figure 1) revealed that recurrence rates were higher in patients whose conduction phenotype included CF (8 of 36 [22.2%]) than in those without CF (4 of 44 [9.1%]; log-rank *P* = .090). This pattern aligns with the double group’s signal, in which 11 of 15 patients (73%) contained CF and contributed 4 of the 5 observed recurrences. The lower recurrence rates in triple and quadruple categories—despite all containing CF—may reflect the protective effect of adjunctive ablation, which was applied at substantially higher rates in these groups (36% and 57%, respectively, vs 20% in double). These findings are consistent with the hypothesis that CF may mark a recurrence-prone substrate, which warrants prospective evaluation in larger cohorts.

### Clinical implications

Our findings suggest that COHERENT vector-flow mapping may assist in characterizing the atrial substrate before ablation. However, whether phenotype-guided ablation strategies improve clinical outcomes was not tested in this study. Patients with intermediate phenotype burden (the double category) had the highest 24-month recurrence rate (33.3%) yet received the lowest rate of adjunctive ablation beyond PVI, suggesting that this group may particularly benefit from more aggressive upfront substrate-modifying strategies in future studies. The BBS-only and quadruple categories, which converged on the same magnitude of borderline stroke/TIA association via apparently distinct mechanisms, may warrant closer thromboembolic-risk assessment. These hypotheses require prospective validation before clinical implementation. Future multicenter studies are required to validate these phenotypes, assess their reproducibility, and determine whether phenotype- or burden-guided ablation strategies improve arrhythmia control and inform thromboembolic-risk assessment.

### Limitations

This single-center observational study included only 80 patients, of whom 12 experienced arrhythmia recurrence and 6 had previous stroke/TIA, limiting generalizability. The small number of events distributed across 8 categories, with zero events in the normal reference group, resulted in wide CIs; we used Firth penalized logistic regression to obtain stable estimates when group sizes were small. Phenotype assignment depended on expert visual interpretation of vector-flow maps by 2 blinded electrophysiologists; although disagreement was less than 5%, Cohen’s kappa was not prospectively recorded, and automated classification algorithms would enhance reproducibility in future studies.

The observational, nonrandomized design meant that adjunctive ablation lesions were applied at the operator’s discretion, which may confound the relationship between conduction phenotypes and arrhythmia recurrence. COHERENT mapping was performed only at baseline, without assessment of dynamic changes in conduction patterns after ablation. Rhythm monitoring relied on periodic 12-lead ECGs, intermittent Holter or patch recordings, and symptom-driven assessment without continuous implantable loop recorder monitoring, potentially underestimating asymptomatic recurrences and silent cerebrovascular events. The cohort also excluded 4 patients who required coronary sinus pacing during the mapping procedure, because such pacing produces a nonphysiological activation pattern bypassing the normal Bachmann’s bundle–mediated interatrial conduction and is unsuitable for COHERENT vector-flow mapping; the generalizability of our findings to patients with severe sinus node dysfunction or atrioventricular block requiring atrial pacing remains to be established.

Future research should validate these findings in larger, multicenter cohorts and develop automated algorithms for phenotype classification. Randomized trials comparing phenotype- or burden-guided ablation strategies with standard PVI are needed to determine whether tailoring lesion sets improves outcomes, particularly for the intermediate-burden double phenotype identified in our analysis.

## Conclusion

Sinus-rhythm COHERENT mapping delineated 8 MECE phenotype categories that captured a graded continuum of atrial cardiomyopathy. The intermediate-burden double category was the only group with a nominally significant association with 24-month arrhythmia recurrence, with adjunctive ablation rates inversely related to phenotype burden, suggesting an “intermediate substrate trap” that may particularly benefit from more aggressive upfront substrate-modifying strategies. BBS only and quadruple converged on the same magnitude of borderline thromboembolic association via distinct focal and global mechanisms. These findings provide mechanistic insight into atrial cardiomyopathy and generate hypotheses for phenotype- or burden-guided ablation and thromboembolic-risk assessment strategies that require validation in larger, multicenter studies.

## Disclosures

The authors have no conflicts of interest to disclose.

## Figures and Tables

**Supplementary Figure:**
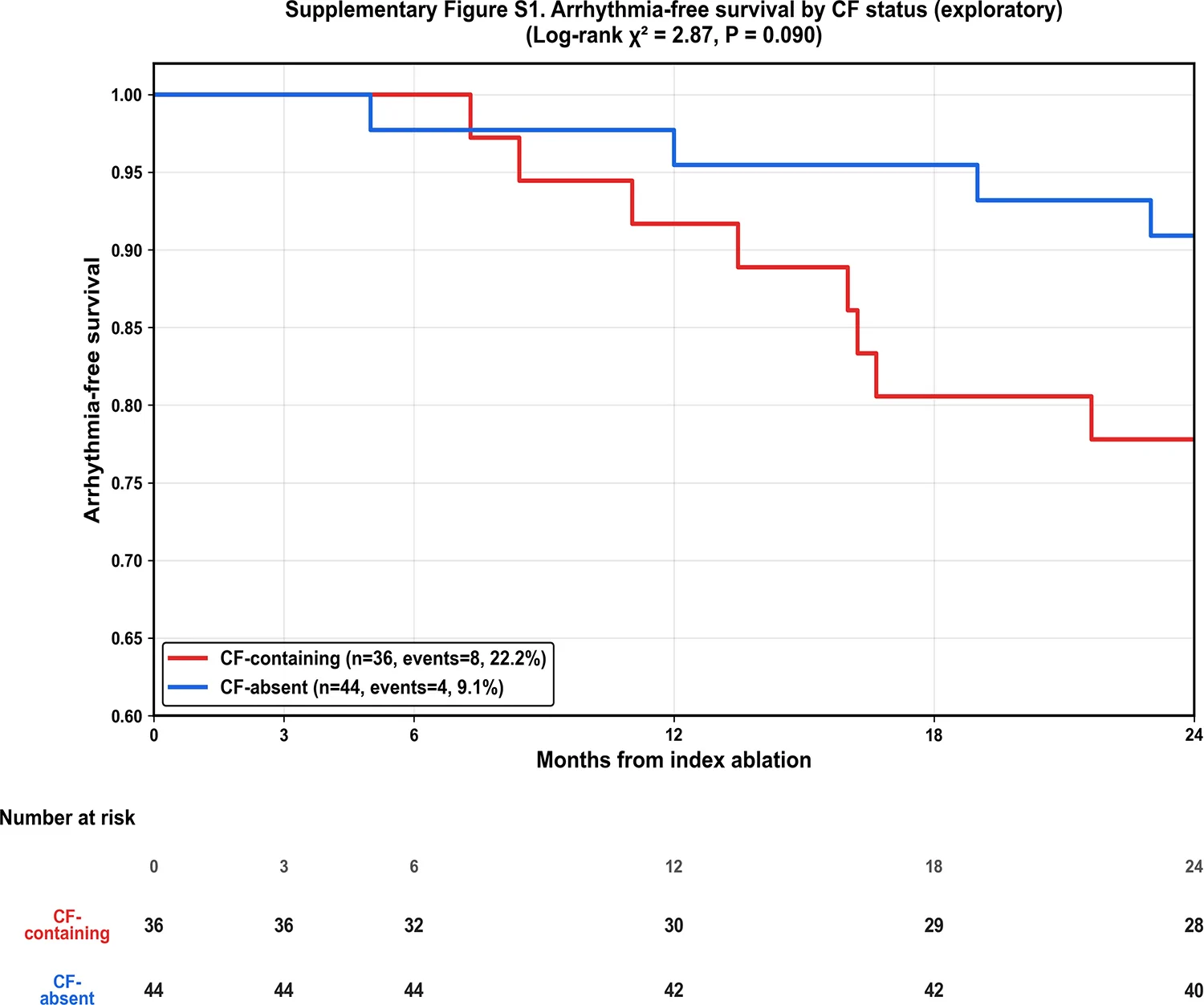


## Footnotes

Appendix Supplementary data associated with this article can be found in the online version at https://doi.org/10.1016/j.hroo.2026.06.012.
