# Supplementary material for: Sinus-rhythm conduction phenotyping using vector-flow mapping in first-time atrial fibrillation ablation: Associations with structural remodeling and thromboembolic risk
Source: Heart Rhythm O2. 2026 Jul 18;7(9):1667–79. doi: 10.1016/j.hroo.2026.06.012 (PMC13594457; doi:10.1016/j.hroo.2026.06.012)
Supplement: Supplementary file 1 [file mmc1.docx]

**Supplementary Tables**

Sinus Rhythm Conduction Phenotyping Using Vector Flow Mapping in First-Time Atrial Fibrillation Ablation: Associations with Structural Remodeling and Thromboembolic Risk

**Supplementary Table S1.**

**Patient flow accounting for all 107 patients screened.**

| **Step** | **n excluded** | **n remaining** | **Reason for exclusion** |
| --- | --- | --- | --- |
| Initial screening | — | 107 | Consecutive patients with symptomatic AF referred for catheter ablation (June 1, 2020 – September 30, 2021) |
| Exclusion 1 | 23 | 84 | Patients with previous AF ablation (second or subsequent session) |
| Exclusion 2 | 4 | 80 | Patients requiring coronary sinus pacing during the mapping procedure (non-physiologic activation pattern bypassing Bachmann’s bundle, precluding meaningful interpretation of COHERENT vector flow patterns) |
| Final analytic cohort | — | 80 | All 80 patients completed 24-month follow-up; no patients were lost to follow-up |

*AF = atrial fibrillation. The COHERENT vector flow mapping algorithm reconstructs continuous activation patterns based on the assumption of physiological sinus rhythm propagation; coronary sinus pacing reverses the normal cranio-caudal activation gradient and bypasses Bachmann’s bundle-mediated interatrial conduction, rendering the resulting vector flow patterns non-interpretable within the predefined classification framework.*

**Supplementary Table S2.**

**Frequency of the 16 mutually exclusive phenotype combinations (N = 80).**

| **Phenotype combination** | **Burden (# of abnormal phenotypes)** | **n** |
| --- | --- | --- |
| Normal | 0 | 23 |
| BBS | 1 | 7 |
| DF | 1 | 5 |
| DF+BBS | 2 | 0 |
| CF | 1 | 7 |
| CF+BBS | 2 | 4 |
| CF+DF | 2 | 4 |
| CF+DF+BBS | 3 | 3 |
| CD | 1 | 5 |
| CD+BBS | 2 | 2 |
| CD+DF | 2 | 2 |
| CD+DF+BBS | 3 | 0 |
| CD+CF | 2 | 3 |
| CD+CF+BBS | 3 | 2 |
| CD+CF+DF | 3 | 6 |
| CD+CF+DF+BBS | 4 | 7 |

*Burden = number of abnormal phenotypes (CD, CF, DF, BBS) present. The Normal category (burden = 0) has all four phenotypes absent. The 16 combinations sum to N = 80 patients. For statistical modeling in the main analysis, these 16 cells were collapsed into the eight MECE phenotype categories (Normal, four single-phenotype groups, Double, Triple, Quadruple) presented in Tables 1–5. CD = conduction delay; CF = congested flow; DF = disrupted flow; BBS = Bachmann’s bundle split.*

**Supplementary Table S3.**

**MECE phenotype category distribution by atrial fibrillation type (Paroxysmal vs Persistent).**

| **MECE phenotype category** | **Paroxysmal AF**  **(n=54)** | **Persistent AF**  **(n=26)** |
| --- | --- | --- |
| Normal | 20 (37.0) | 3 (11.5) |
| CD only | 4 (7.4) | 1 (3.8) |
| CF only | 5 (9.3) | 2 (7.7) |
| DF only | 3 (5.6) | 2 (7.7) |
| BBS only | 7 (13.0) | 0 (0.0) |
| Double | 9 (16.7) | 6 (23.1) |
| Triple | 3 (5.6) | 8 (30.8) |
| Quadruple | 3 (5.6) | 4 (15.4) |

*Data are presented as n (%). The eight mutually exclusive (MECE) phenotype categories are cross-tabulated against atrial fibrillation type (Paroxysmal vs Persistent). Persistent AF was over-represented in the highest-burden categories (Triple 30.8%, Quadruple 15.4%) compared with Paroxysmal AF (Triple 5.6%, Quadruple 5.6%). AF = atrial fibrillation; CD = conduction delay; CF = congested flow; DF = disrupted flow; BBS = Bachmann's bundle split.*

**Supplementary Table S4.**

**Multinomial logistic regression: additional predictors of MECE phenotype categories (vs Normal as reference).**

| **Phenotype (vs Normal)** | **P-wave duration (per 1 ms)** | **CHA₂DS₂-VASc (per 1 point)** | **Age (per 10 years)** | **Female sex (vs male)** | **BMI (per 1 kg/m²)** |
| --- | --- | --- | --- | --- | --- |
| CD only | 1.03 (0.97–1.09) | 1.14 (0.58–2.22) | 1.08 (0.51–2.29) | 1.38 (0.19–9.83) | 1.00 (0.74–1.35) |
| CF only | 0.93 (0.85–1.01) | 1.32 (0.74–2.35) | 1.98 (0.85–4.62) | 1.22 (0.22–6.73) | 0.99 (0.76–1.29) |
| DF only | 1.09 (1.00–1.19) | 0.47 (0.18–1.20) | 1.20 (0.54–2.63) | 0.61 (0.09–4.37) | 1.20 (0.94–1.53) |
| BBS only | 1.01 (0.96–1.07) | 1.40 (0.79–2.50) | 1.09 (0.57–2.12) | 1.22 (0.22–6.73) | 1.11 (0.87–1.41) |
| Double | 1.03 (0.98–1.07) | 1.10 (0.70–1.74) | 1.48 (0.84–2.60) | 0.46 (0.12–1.77) | 1.08 (0.89–1.31) |
| Triple | 1.05 (0.98–1.12) | 1.27 (0.77–2.09) | 1.20 (0.67–2.14) | 0.52 (0.12–2.29) | 1.15 (0.94–1.41) |
| Quadruple | 1.01 (0.94–1.09) | 2.06 (1.11–3.84) | 1.55 (0.72–3.33) | 0.69 (0.13–3.79) | 1.15 (0.91–1.45) |

*Each cell shows odds ratio with 95% confidence interval (OR [95% CI]) from a multinomial logistic regression model with the eight MECE phenotype categories as the outcome, Normal as the reference, and each candidate predictor entered individually in a separate single-predictor model. P-wave duration was available in 58 of 80 patients (22 missing); other predictors had complete data. Quadruple CHA₂DS₂-VASc OR 2.06 (1.11–3.84) reached statistical significance.*

**Supplementary Table S5.**

**Twelve-month arrhythmia recurrence by MECE conduction phenotype category (Firth penalized logistic regression, secondary analysis).**

| **Phenotype** | **n** | **Events (%)** | **Firth OR (95% CI)** | **p-value** |
| --- | --- | --- | --- | --- |
| Normal (reference) | 23 | 0 (0.0) | Reference | — |
| CD only | 5 | 0 (0.0) | 4.27 (0.06–306.01) | 0.505 |
| CF only | 7 | 2 (28.6) | 21.37 (0.81–566.36) | 0.067 |
| DF only | 5 | 0 (0.0) | 4.27 (0.06–306.01) | 0.505 |
| BBS only | 7 | 2 (28.6) | 21.37 (0.81–566.36) | 0.067 |
| Double | 15 | 3 (20.0) | 13.16 (0.59–295.70) | 0.105 |
| Triple | 11 | 1 (9.1) | 6.71 (0.23–195.91) | 0.269 |
| Quadruple | 7 | 0 (0.0) | 3.13 (0.05–206.79) | 0.593 |

*Twelve-month follow-up analysis (8 events / 80 patients = 10.0%) provided as a secondary comparison to the primary 24-month analysis (Table 4). Firth penalized likelihood was used because the Normal reference group had zero events. Likelihood ratio test p = 0.261; c-statistic = 0.835. None of the categories reached statistical significance at 12 months; the Double category became significant at 24-month follow-up (Table 4).*

**Supplementary Table S6.**

**Sensitivity analysis by MECE phenotype category, excluding patients with pre-existing cardiomyopathy (n = 67).**

| **Variable** | **Normal**  **(n=20)** | **CD only**  **(n=5)** | **CF only**  **(n=6)** | **DF only**  **(n=5)** | **BBS only**  **(n=7)** | **Double**  **(n=12)** | **Triple**  **(n=9)** | **Quadruple**  **(n=3)** |
| --- | --- | --- | --- | --- | --- | --- | --- | --- |
| Age, mean±SD (yrs) | 62.3±15.3 | 63.2±17.8 | 71.5±9.6 | 64.8±4.7 | 63.4±11.0 | 68.2±13.2 | 64.0±10.8 | 66.3±18.5 |
| Female sex, n (%) | 12 (60.0) | 3 (60.0) | 4 (66.7) | 2 (40.0) | 4 (57.1) | 4 (33.3) | 4 (44.4) | 2 (66.7) |
| Hypertension, n (%) | 8 (40.0) | 4 (80.0) | 4 (66.7) | 2 (40.0) | 7 (100.0) | 6 (50.0) | 7 (77.8) | 3 (100.0) |
| LVEF, mean±SD (%) | 67.1±5.7 | 68.4±5.7 | 65.5±4.8 | 66.0±7.3 | 69.7±6.7 | 64.7±8.6 | 67.8±8.8 | 67.0±14.0 |
| LAVI, mean±SD (mL/m²) | 28.3±11.3 | 24.0±5.5 | 37.3±14.6 | 37.2±3.3 | 26.0±6.6 | 34.8±12.7 | 37.8±7.0 | 43.0±10.4 |
| NT-proBNP, median [IQR] (pg/mL) | 141 [46–438] | 16 [15–125] | 246 [181–534] | 489 [166–666] | 129 [98–355] | 278 [123–646] | 406 [384–657] | 379 [226–1320] |
| 12-month AF/AT recurrence, n (%) | 0 (0.0) | 0 (0.0) | 2 (33.3) | 0 (0.0) | 2 (28.6) | 2 (16.7) | 0 (0.0) | 0 (0.0) |
| 24-month AF/AT recurrence, n (%) | 0 (0.0) | 1 (20.0) | 2 (33.3) | 0 (0.0) | 2 (28.6) | 4 (33.3) | 0 (0.0) | 0 (0.0) |
| Prior stroke/TIA, n (%) | 0 (0.0) | 0 (0.0) | 0 (0.0) | 0 (0.0) | 2 (28.6) | 0 (0.0) | 1 (11.1) | 1 (33.3) |

*Data are presented as mean ± standard deviation, n (%), or median [interquartile range]. Thirteen patients with pre-existing cardiomyopathy (tachycardia-induced, n = 5; hypertrophic, n = 3; dilated, n = 2; ischemic, n = 1; amyloidosis, n = 1; HCM with suspected amyloidosis, n = 1) were excluded, yielding n = 67. The eight MECE phenotype categories (Normal, four single-phenotype groups, Double, Triple, Quadruple) are preserved in this sensitivity analysis. Recurrence cumulative event counts at 12 and 24 months are tabulated for each category; the small subgroup sizes preclude formal hypothesis testing within this sensitivity cohort. LAVI = left atrial volume index; LVEF = left ventricular ejection fraction; NT-proBNP = N-terminal pro-B-type natriuretic peptide; TIA = transient ischemic attack.*

**Supplementary Figure**

**Supplementary Figure S1.**

**Arrhythmia-free survival by CF status (exploratory).**

Kaplan–Meier curves comparing 24-month arrhythmia-free survival between patients whose conduction phenotype contained CF (n = 36, 8 events, 22.2%; red) and those without CF (n = 44, 4 events, 9.1%; blue). The log-rank test yielded χ² = 2.87 (P = 0.090). Number at risk is shown below the time axis. This exploratory subgroup analysis is consistent with the hypothesis that the CF pattern may contribute as a common substrate signal to recurrence spanning CF only and CF-containing multi-phenotype categories. CF = congested flow.
